# Historical grassland desertification changes in the Horqin Sandy Land, Northern China (1985–2013)

**DOI:** 10.1038/s41598-017-03267-x

**Published:** 2017-06-07

**Authors:** Jinya Li, Bin Xu, Xiuchun Yang, Zhihao Qin, Lina Zhao, Yunxiang Jin, Fen Zhao, Jian Guo

**Affiliations:** 1 State Key Laboratory of Urban and Regional Ecology, Research Center for Eco-Environmental Sciences, Chinese Academy of Sciences, Beijing, 100085 P.R. China; 2 Key Laboratory of Agri-informatics of the Ministry of Agriculture, Institute of Agricultural Resources and Regional Planning, Chinese Academy of Agricultural Sciences, Beijing, 100081 P.R. China; 3 Institute of Botany, Chinese Academy of Sciences, Beijing, 100093 P.R. China; 4 Key Laboratory of Ecosystem Network Observation and Modeling, Institute of Geographic Sciences and Natural Resources Research, Chinese academy of Science, Beijing, 100086 China

## Abstract

Since rural reforms in the 1980s, both the state and local governments of China have devoted great efforts to combating desertification through a number of eco-environmental restoration campaigns, resulting in burgeoning contention at all levels of government and sparking public concern. Monitoring and accurately assessing the statuses and trends of grassland desertification are important for developing effective restoration strategies. The Horqin Sandy Land (HSL), a very typical desertified grassland (DG) with better hydrothermal conditions among sandy lands in north China, was recently selected (1985–2013) to assess the spatiotemporal dynamic performances of grassland desertification before and after implementing restoration projects. Landsat images (TM/ETM+/OLI), field investigations and expert review were integrated to form a classification scheme for the HSL. Then, spectral mixture analysis and the decision-tree method were used to extract bare-sand ratios and vegetation cover fraction dynamics. A favourable phenomenon of DG was seen to be reversed in an accelerated pace during 2001–2013, despite challenge from both climatic and anthropogenic factors. However, overexploitation of grassland (especially for farming) and ground water for irrigation has led to remarkable decreases in the ground water level in recent decades, which should be highly concerning regarding the formulation of restoration campaigns in the sandy land.

## Introduction

Desertification is a type of land degradation that occurs in arid, semi-arid, and sub-humid regions due to the synthetic action of climatic variations and human activities^1^. Governments around the world have publicly regard desertification as a serious threat (approximately 32% of the world’s population, 67% of countries, and 40% of the land area worldwide are directly or indirectly affected by desertification) and pledged to reduce the rate of land degradation since the implementation of the Plan of Action to Combat Desertification (PACD)^2^. However, little progress has been made, and the Parties to the United Nations Convention to Combat Desertification (UNCCD) still have no accurate estimates of the extent of desertification^3, 4^. Desertification monitoring and assessment (M & A) must be conducted if the Parties to the UNCCD are to have reliable information that supports monitoring the effectiveness of their activities^5^.

Remote sensing for monitoring land degradation was selected as one of three topics to be discussed at the fourth Drylands Deserts and Desertification “DDD” conference held at Ben Gurion University in November 2014^4, 6^. New ideas and scientific support for combating desertification are urgently needed because the challenges involved in combating desertification are still considerable^6^. In many studies, desertification monitoring has been conducted based on changes in vegetation, mainly detected by the NDVI (normalized differential vegetation index), which is derived from red and near-infrared wavebands^7^. Vegetation indices (VIs) are robust, provide an empirical method for monitoring the dynamics of vegetation, and are easily obtained. However, establishing a direct relationship between VIs and grassland desertification is difficult because VIs are vulnerable to soil background differences and other factors, such as precipitation fluctuations^8–11^. Additionally, in China, commonly used methods include visual interpretation and field investigation, which are expensive, labour intensive, and often limited at spatial-temporal scales^12, 13^. Given that pixels in remote sensing images are usually mixed, especially in arid and semiarid environments, extracting information at the sub-pixel scale is important. Spectral mixture analysis (SMA), which is a subpixel classification method, has been promoted as an efficient method for deriving coverage information from multispectral/hyperspectral images^14–16^. However, the application of SMA in desertification research requires further investigation.

Grasslands serve important and global roles because they cover approximately one fifth of the terrestrial land surface and balance the global carbon (C) budget; however, grasslands are especially vulnerable to changes^17^. Given a population of more than 1.3 billion people and limited farmland area, efficient and sustainable grassland management is a survival issue for China because China contains the second largest area of grassland in the world (approximately 400 million ha)^18^. However, 90% of China’s available natural grasslands exhibit varying degrees of degradation, with most grasslands being distributed in 498 counties in 18 provinces in northwest, north, and northeast China, all of which are poverty-stricken regions with fragile ecosystems.

The HSL is the largest sandy land and has the best hydrothermal conditions among the sandy lands in China but faces high anthropogenic pressure under rapid economic development, especially animal husbandry and reclamation. It is very helpful to study the dynamic changes in grassland desertification under the interactions between climate change and anthropogenic factors for understanding the man-earth relationship and for developing effective solutions. The specific objectives of this study were (1) to design a grassland desertification grading system applicable to HSL; (2) to investigate the suitability of Landsat images and SMA methods for grassland desertification monitoring; and (3) to monitor and analyse the dynamics of grassland desertification during the previous 30 years in the sandy land.

## Results

### Status and spatial distribution of grassland desertification during four periods

**Figure 1:**
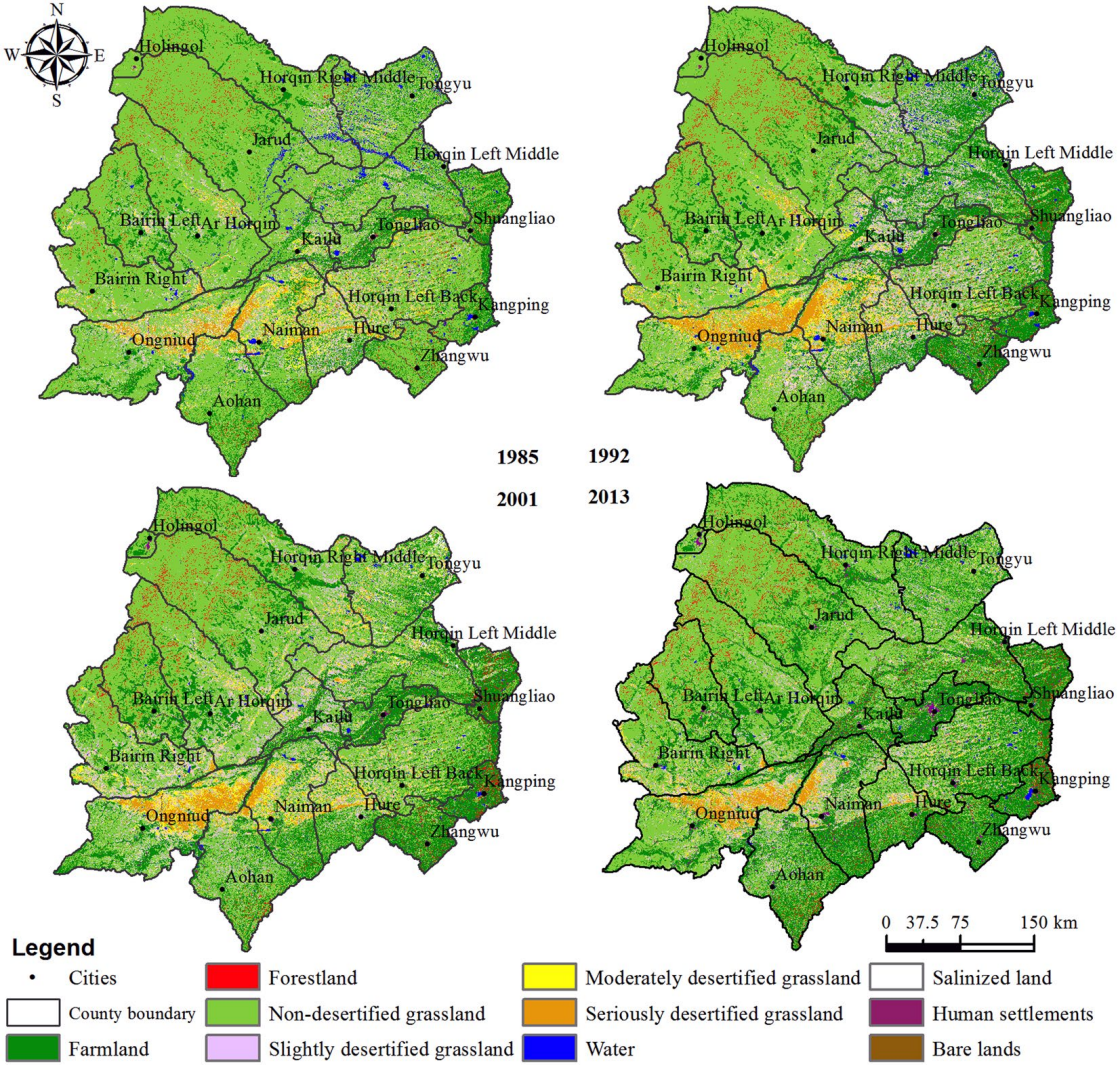
Status and spatial distribution of grassland desertification over four periods in HSL. The maps were generated by ArcGIS software (Version 10.0, ESRI, Redlands, CA, USA, http://www.esri.com/).

The status and spatial distribution of grassland desertification during four periods in the HSL are shown in Fig. 1, and the statistical results are shown in Table 1. The results show that the landcover in the study area is mainly grassland, which accounts for 70% of the entire territory. Non-, slightly, moderately, and severely DG account for 55%, 9%, 4%, and 3%, respectively, of the total grassland area. The total DG area covered approximately 15% of the HSL and exhibited an increase of 7,817 km^2^ from 1985 to 1992 before gradually decreasing to 16,559 km^2^ in 2013. The distribution of DG in the HSL represents obvious zonality. Moderately desertified grassland (MDG) and severely desertified grassland (SeDG) are mainly distributed southwest of HSL, where Ongniud, Naiman, Hure, and Aohan are involved in the administrative division. In addition, SeDGs are mainly located in the triangle region that is formed by Wufendi-Wudan-Wuduntaohai in Ongniud and the Xar Moron and Jiaolai Rivers.

**Table 1:** Areas and proportions of each class over four periods in the Horqin Sandy Land (Area: km^2^, Proportion: % of Horqin Sandy Land).

Land Type	1985		1992		2001		2013	
	*Area*	*Proportion*	*Area*	*Proportion*	*Area*	*Proportion*	*Area*	*Proportion*
SlDG	10,431	7.55	14,618	10.59	14,923	10.81	10,410	7.55
MDG	5,374	3.89	6,749	4.89	6,794	4.92	3,265	2.37
SeDG	3,087	2.24	5,342	3.87	3,765	2.73	2,883	2.09
Total DG	18,892	13.68	26,709	19.34	25,482	18.45	16,559	12.00
Non-DG	90,894	65.81	72,659	52.62	71,855	52.03	70,171	50.87
Total Grassland	109,786	79.49	99,368	71.96	97,337	70.48	86,730	62.87
Farmland	20,932	15.16	31,677	22.94	34,236	24.79	44,098	31.97
Forestland	2,306	1.67	2,949	2.14	3,122	2.26	3,520	2.55
Water	2,579	1.87	2,115	1.53	891	0.65	815	0.59
Salinized Land	1,370	0.99	780	0.56	928	0.67	246	0.18
Human Settlement	453	0.33	683	0.49	1,115	0.81	1,993	1.44
Bare Land	679	0.49	522	0.38	467	0.34	548	0.40
Total Non-Grassland	28,320	20.51	38,727	28.04	40,759	29.52	51,221	37.13

SlDG: Slightly desertified grassland, MDG: Moderately desertified grassland, SeDG: Severely desertified grassland, DG: Desertified grassland.

### Dynamic spatio-temporal changes in grassland desertification over four periods

**Figure 2:**
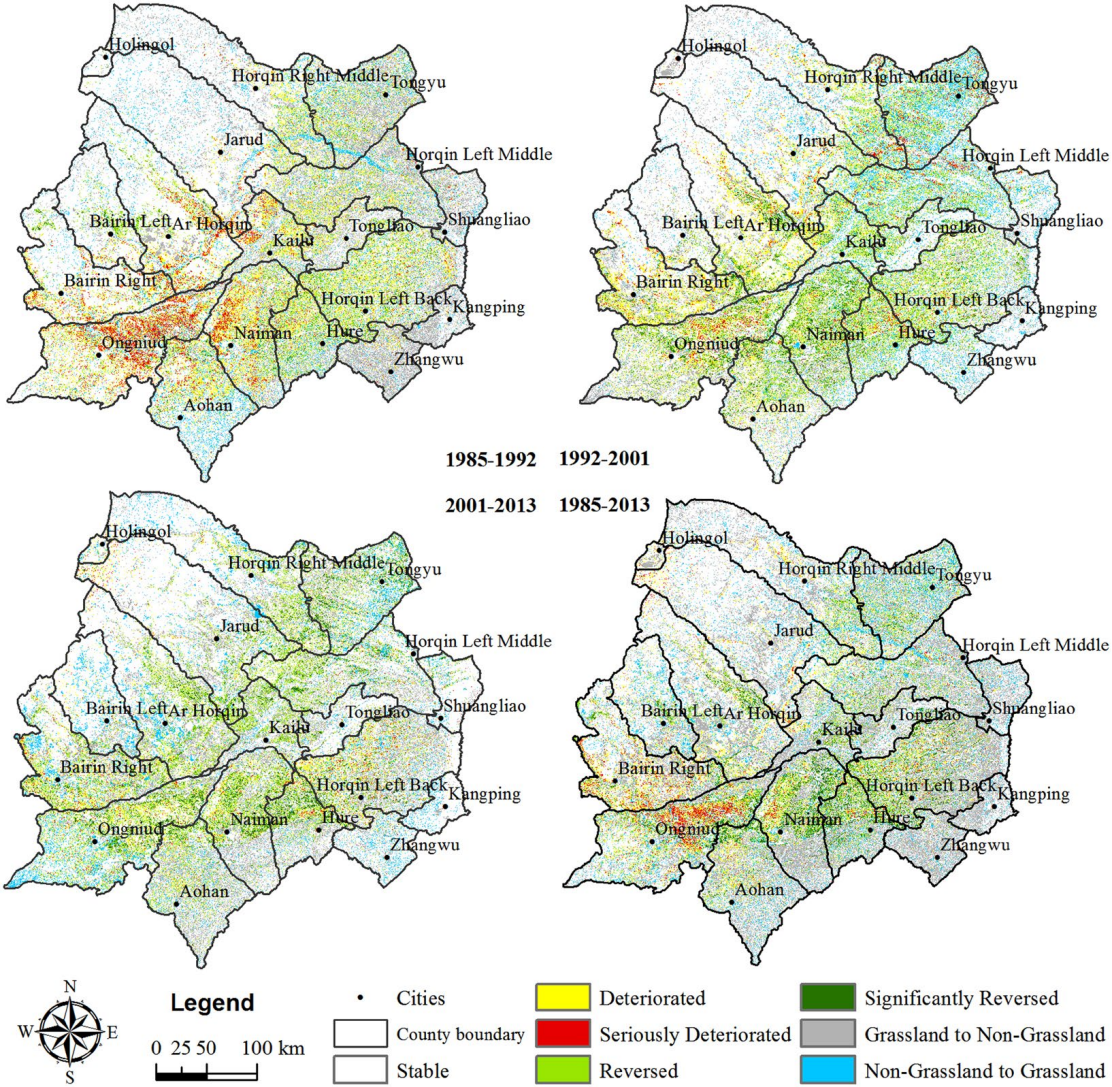
Dynamic spatio-temporal changes in grassland desertification over four periods. The maps were generated by ArcGIS software (Version 10.0, ESRI, Redlands, CA, USA, http://www.esri.com/).

Figure 2 was generated through raster calculation in ArcGIS software (Version 10.0, ESRI, Redlands, CA, USA, http://www.esri.com/) and change detection and decision tree in ENVI software (Version 4.7, Exelis Visual Information Solutions Inc., Boulder, Colorado, USA, http://www.exelisvis.com/) to represent the dynamic changes over four periods. The statistical results are shown in Table 2. Through further processing of DG data and other types of land data from various periods, an annual rate was obtained (Table 3). From 1985 to 1992, the DGs with ‘Reversed’ and ‘Significantly Reversed’ degrees were mainly distributed in the central part of the Bairin Left Banner and the northern part of the Bairin Right Banner. However, the desertification ‘Deteriorated’ and ‘Seriously Deteriorated’ grasslands were mainly distributed in the northeast regions of Ongniud, the northwest regions of Naiman and the west regions of Kailu County. During this period, the annual changes of all three DG levels were positive, especially for the SeDGs (10.44%). From 1992 to 2001, the ‘Reversed’ and ‘Significantly Reversed’ degrees were mainly interspersed among the Naiman Banner, the northern part of the Aohan Banner and the eastern part of the Ar Horqin Banner, while the ‘Deteriorated’ and ‘Seriously Deteriorated’ degrees were mainly distributed in the mid-eastern part of the Ongniud Banner and the northeast part of the Hure Banner. During this period, slightly desertified grassland (SlDG) and MDG continued to expand, but the range was reduced while SeDG and Total-DG began to shrink, suggesting an overall reversion in grassland desertification. From 2001 to 2013, the grassland desertification in HSL was mainly ‘Reversed’ and ‘Significantly Reversed’ (with annual reduction rates of 2.52%, 4.33% and 1.95% in SlDG, MDG and SeDG, respectively), except for some ‘Deteriorated’ and ‘Seriously Deteriorated’ DGs in the Horqin Left Back Banner, the central part of the Hure Banner and the Bairin Right Banner. During all three periods, the rapidly expanding farmland and human settlement areas and continuously decreasing open-water surface area demonstrated the growing pressure of land use and the potential risk of another grassland desertification.

**Table 2:** Spatio-temporal dynamic changes over different periods (area: km^2^; proportion: %).

Change Direction	1985–1992		1992–2001		2001–2013		1985–2013	
	*Area*	*Proportion*	*Area*	*Proportion*	*Area*	*Proportion*	*Area*	*Proportion*
Deteriorated	11,291	8.17	8,890	6.43	5,456	3.95	6,963	5.04
Seriously Deteriorated	4,019	2.91	2,308	1.67	1,142	0.83	2,488	1.80
Total Deteriorated	15,311	11.08	11,198	8.11	6,598	4.78	9,451	6.84
Reversed	5,510	3.99	11,400	8.25	11,256	8.15	6,612	4.79
Significantly Reversed	1,165	0.84	3,442	2.49	4,017	2.91	3,814	2.76
Total Reversed	6,675	4.83	14,842	10.74	15,273	11.06	10,425	7.55
Grassland to Non-Grassland	17,527	12.69	11,071	8.01	18,199	13.17	32,174	23.29
Non-Grassland to Grassland	7,121	5.15	9,039	6.54	7,723	5.59	9,258	6.70
Stable	91,516	66.24	92,002	66.60	90,357	65.40	76,842	55.62

**Table 3:** Annual change rates for each major category in the Horqin Sandy Land over different periods (units: %).

Annual rate	SlDG	MDG	SeDG	Total DG	Non-DG	Farmland	Forestland	Water	Salinized Land	Human settlement	Bare Land
1985–1992	5.73	3.66	10.44	5.91	−2.87	7.33	3.98	−2.57	−6.15	7.25	−3.30
1992–2001	0.23	0.07	−3.28	−0.51	−0.12	0.90	0.65	−6.43	2.11	7.03	−1.17
2001–2013	−2.52	−4.33	−1.95	−2.92	−0.20	2.40	1.06	−0.71	−6.12	6.56	1.45

Positive values represent an increase, and negative values represent a decrease.

## Discussion

The HSL is located in the agriculture and pasture interlaced zone of northern China. The harsh natural environment and backward economic conditions make the eco-environment here more sensitive and vulnerable. The landscape, vegetation, soil, and particularly the land use in this zone express some transitivity and volatility, which are sensitive to human activities. Mismanagement and climate variations may lead to unsustainable development and soil degradation, especially in arid and semi-arid regions such as Horqin^19, 20^.

**Figure 3:**
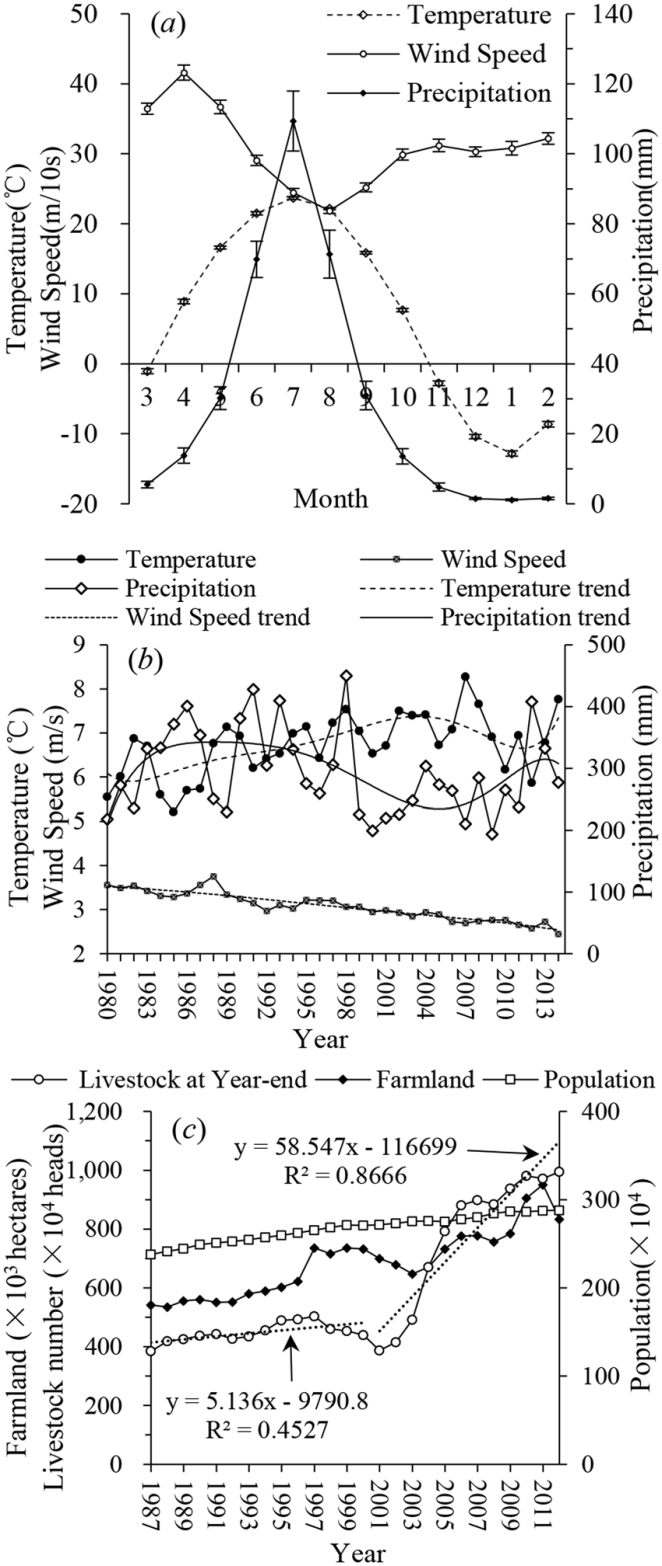
Trends of climatic and human-related factors in the study area. The meteorological data are averaged values from the weather stations in Ongniud, Naiman, Jarud, Barin left and Tongliao, and the socioeconomic data are the averaged values of the statistic almanac data from these banners. (**a**) is the monthly averaged value and its standard deviation from 1980 to 2014. (**b**) and (**c**) are the annual average values.

In arid and semi-arid regions, precipitation is always the most limiting factor. The average temperature increased before 2004 and then decreased (Fig. 3b). The annual precipitation varied following an M shape trend, and low amounts of rainfall occurred after 1998. The upward trend in the annual temperature and the opposite trend in annual precipitation made the environment unsuitable for vegetation growth and the recovery of desertification. Furthermore, the uneven distribution of precipitation throughout the year also resulted in limitations. Overall, 70.87% of the yearly precipitation occurred during the three summer months, while only 3.24% and 2.64% of the total yearly precipitation occurred during the spring and winter months (Fig. 3a). The temperature changes followed the same distribution as precipitation. The average annual wind speed generally decreased, exhibiting an opposite distribution to precipitation. During the grass-withering period, which includes low vegetation coverage but strong winds, the bare surfaces are vulnerable to wind erosion, particularly during the spring as temperature and evapotranspiration increase. The long drought and windy season, uneven precipitation distribution and global warming cause this eco-environment in the HSL to be fragile and sensitive^21^.

Grassland desertification is a result of the interactions between climate change and anthropogenic factors. Climate change serves as a background to the occurrence and development of desertification and guides the evolution of desertification over long temporal and spatial scales. If the harsh environment provides the background for desertification, human activities provide disturbance factors. With socioeconomic development and the burgeoning demands of people for a better life, land is facing more anthropogenic pressure as an energy source. However, at specific times and locations, human activities can also influence the process of desertification and even inhibit or reverse desertification processes through particular behaviours or measures based on understanding the mechanisms of desertification. Facing the continued worsening of grassland desertification, state and local governments have made great efforts to reduce grassland desertification and have created a series of national ecological projects, such as the Grain for Green Project (1999–present), the Beijing and Tianjin Sandstorm Source Treatment Project (2001–2010), and the Three-North Shelterbelt Project (1978–present). In particular, the grazing ban (2003–present), which prevents livestock from trampling or eating the grass in grassland areas, has effectively reduced grassland desertification^22^. In the Naiman banner, which contains the main DG with the Ongniud banner in HSL, by 2012, a total of RMB 400 million yuan from these projects had been invested to cover 3497 km^2^, including 303 km^2^ for the Three-North Shelterbelt Project, 711 km^2^ for the Grain for the Green Project, 1933 km^2^ for the Restore Pasturage to Natural Grassland Project and 550 km^2^ for the Watershed Project^23^. Figure 3 shows the continuous population growth and fluctuating increase in the year-end number of livestock, which significantly increased following 2001 and should be a negative factor for reversing desertification. However, as shown in this paper and in similar studies^20^, desertification has been reversed in recent decades. The effects of these eco-environmental projects are considered to play important roles in vegetation restoration and desertification reversion^22, 24–28^.

However, the total area of farmland is fluctuating and generally increasing. Large areas of rich grassland have been reclaimed to obtain economic value as grazing has become restricted. In the absence of enough fertile land, some lowland areas among sandy dunes have been expropriated, where the soil is thin with poor structure. Although grazing has been restricted and degraded grasslands have low productivity, herdsmen have compensated by increasing for the number of livestock (Fig. 3c). The groundwater table also decreased linearly^29–31^, setting up the area for the next grassland degradation. To maintain sustainable eco-environmental restoration and the effectiveness of treatment projects, on no account can we ignore the interests of the residents in the affected region^32^.

The relationships between man and earth form the foundation for the survival and progress of humans and the environment. To address this problem, the Reward-compensation Mechanism of Grassland Ecology Protection (2011–present) was created to secure the futures of these eco-environmental projects. RMB 77.36 billion yuan was invested during the “12^th^ Five-year Plan” period (2011–2015), and investment increased during the “13^th^ Five-year Plan” period (2016–2020) according to the Ministry of Agriculture of P.R. China and the Ministry of Finance of P.R. China^33, 34^. A certain effect has been obtained, e.g., decreasing farmland and the inhibition of livestock growth at the end of the year. However, further efforts are still needed to achieve some interactions and synergy between these projects.

## Conclusion

Despite the challenging climate conditions, the monitoring results presented in this paper show that grassland desertification has been in a state of reversion in the last two decades, including rapid reductions in DGs (with annual reduction rates of 2.52%, 4.33%, 1.95% and 2.92% in SlDG, MDG, SeDG and Total-DG, respectively) after 2001. This indicates that the eco-environmental restoration projects are effective and successful. Nonetheless, the overexploitation of grasslands should be considered, especially the falling water tables. As always, there is a trade-off between environment and development; more effort and wisdom are needed for further restoration campaigns in the sandy land.

To improve or develop effective and timely management strategies, rapid and continuous monitoring and assessment of grassland desertification are necessary. In this paper, satellite images were shown to be valuable for historical and large-scale monitoring. However, despite the objective information derived by SMA and its comparative advantages over vegetation cover based methods, several limitations in the accuracy still exist that are based on low spatial and temporal resolution images. Further efforts are still needed to overcome the instantaneous nature of satellite images and to develop more effective methods for information extraction. In addition, the appropriate indicators and grading system are indispensable for desertification monitoring. However, the formulation of a universal grassland desertification monitoring indicator system is a challenging and ambitious task due to the diverse regional desertification characteristics, technological levels, and cognition levels. In this paper, the bare sand ratio was used as a single index to evaluate the grassland desertification. Although it can reduce the impact of precipitation fluctuations and its comparative advantages over vegetation cover-based methods, the treatment method is simple. Whether a single factor can comprehensively reflect the desertification status and how to obtain other direct response desertification information indicators through remote sensing data require further exploration.

## Materials and Methods

### Study area

**Figure 4:**
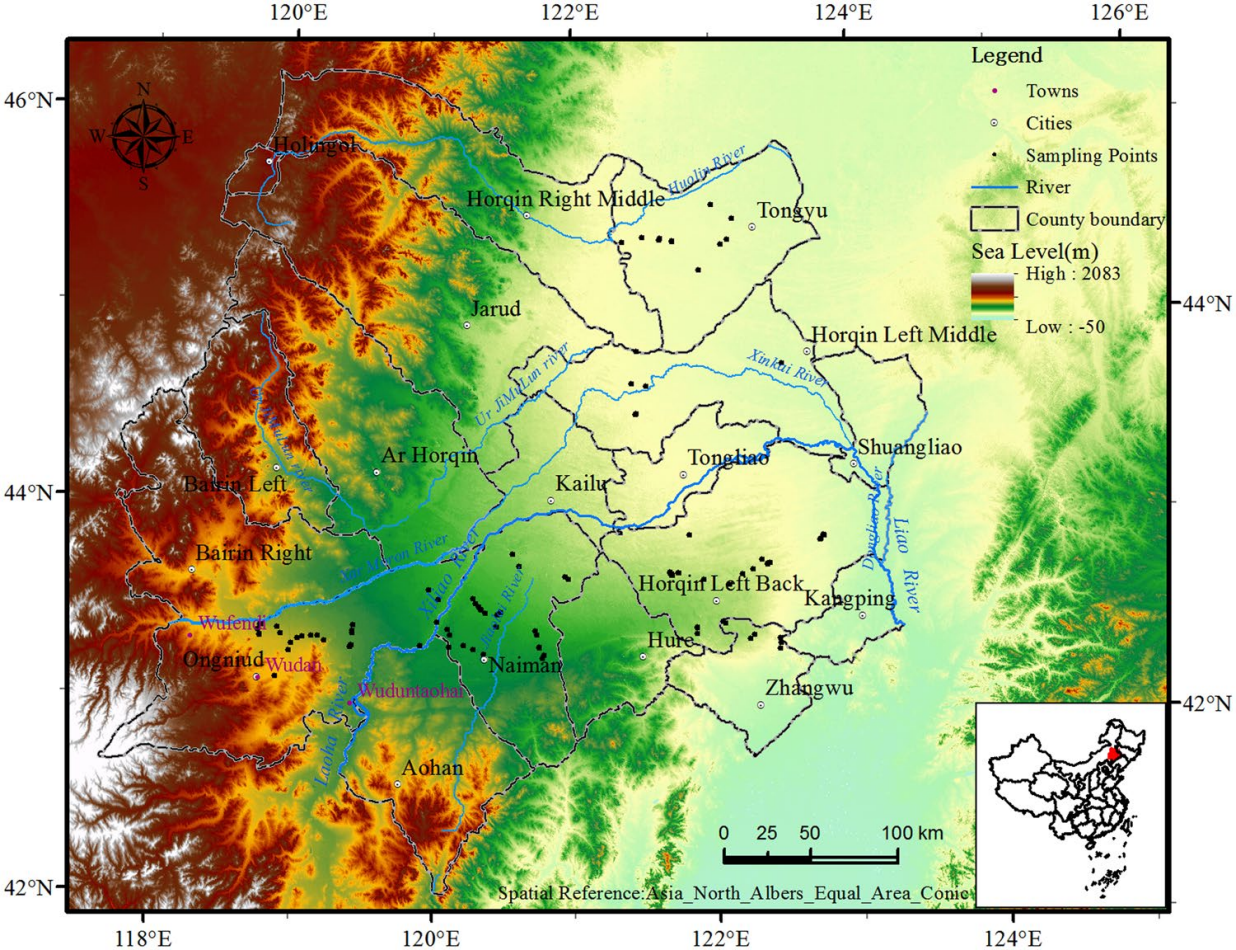
Location of the study area. The maps were generated by ArcGIS software (Version 10.0, ESRI, Redlands, CA, USA, http://www.esri.com/).

The study area is located in the western region of Northeast China and covers a total area of 13.81 × 10^4^ km^2^, from longitude 117°50′E to 124°05′E and latitude 41°40′N to 46°00′N and including 18 administrative counties and cities (Fig. 4). The study area is located in a transition area from the Northeast Plain to the Inner Mongolia Plateau, which extends from the hilly area of the southwestern portion of the Greater Hinggan Mountains to the West Liaohe River Plain. In addition, the study area is in a typical agro-pastoral transition zone and has a temperate continental semi-arid monsoonal climate with an annual mean precipitation of 340–450 mm and an annual mean potential evaporation of 1500–2500 mm. The mean annual temperature was 5.8–6.4 °C (greater than 7 °C in the south and 3–4 °C in the north). The precipitation throughout the year was extremely uneven, primarily occurring during a few summer months (May to September), and the peak precipitation occurred in July or August, which corresponded to the period with the greatest amount of sunlight. This period is called the “hot rainy season”, with 85% of the yearly precipitation and 45% of the yearly sunshine days. The average annual wind speed is 3.4–4.5 m s^−1^, with most windy days and windstorms occurring between March and May, which is before the rainy season.

The landscape is characterized by farmland alternating with natural grassland, and the soils in the area are primarily eolian sandy soils (cover the 84.3% of the total area) and chestnut soils. The native vegetation consists of sparse-tree grasslands, which have been degraded to typical steppe and temperate steppe deserts, and the grassland soils consist of degraded sandy chestnut soils and eolian sandy soils, which are coarse in texture, poor in structure, low in soil nutrient content and weak in water and nutrient preserving capacity, producing an environment that is less favourable for vegetation restoration and desertification reversion.

### Data acquisition and pre-processing

Landsat data are of moderate spatial resolution and offer the longest continuous global record of space-based surface observations, creating a historical archive with unmatched quality, detail, coverage and length and offering a unique opportunity to observe anthropogenic and natural changes at local to global scales^35, 36^. The successful launch of Landsat 8 extended the 40-year Landsat record by at least 5 years, which is very important for global change research^37^. Furthermore, Landsat images are free and widely applied, which reduces costs and allows for large scale use, especially in areas with low economic value, such as grassland desertification areas^38^.

Based on the data quality (cloud coverage of less than 10% or free of clouds), the precipitation data and our goal of analysing the influences of implementing a series of ecological protection and recovery policies for grassland desertification before and after 2002, we selected Landsat Thematic Mapper (TM5) images that were acquired in 1985, 1992 and 2001, a set of Landsat Enhanced Thematic Mapper plus (ETM + 7) images that were acquired in 2001 and Landsat Operational Land Imager (OLI) images that were acquired in 2013 (13 scenes for each period with 30-m resolution). Given the large size of the study area and the frequent occurrence of clouds over the study area, obtaining all images within a given year was difficult; thus, some images were selected that were obtained around the year. These images were acquired during the region’s typical growing season (July and early September, see Supplementary Table S1 for details) and were downloaded from the United States Geological Survey (USGS) web site (http://glovis.usgs.gov) for free.

A geometric correction was applied to the Landsat images of 2001 by selecting ground control points (GCPs) from 1:100,000 topographic maps to maintain the rectification error to less than one pixel. Next, these corrected images were used as base images to warp the images from 1985, 1992 and 2013 using the image-to-image registration method^18, 39^. The nearest neighbour assignment was applied, yielding a RMS error of less than 0.5 pixels. Radiometric calibration was performed to calibrate these images to radiance data via the ENVI software. Then, the radiance images were converted into either band-interleaved-by-pixel (BIP) or band-interleaved-by-line (BIL) interleaves to prepare them for atmospheric correction by using the FLAASH (Fast Line-of-Sight Atmospheric Analysis of Spectral Hypercube) module, which is also commercially available as an add-on to the ENVI software package sold by ITT Exelis Visual Information Solutions^40^. Radiometric calibration was performed to eliminate the errors caused by the sensor, and the FLAASH module was used to eliminate the errors caused by atmospheric absorption and scattering and was used to convert the radiance data of the Landsat images to land surface albedo^41^. Unlike many other atmospheric correction algorithms in which atmospheric parameters rely on synchronous measurements with remote sensing images, FLAASH incorporates the MODTRAN (Moderate Resolution Atmospheric Transmission) radiation transfer code and computes the atmospheric parameters based only on the image spectrum feature. A unique MODTRAN model atmosphere and a selectable aerosol type are combined for different images to ensure acceptable precision^42^. Considering the locations and acquisition times of the images used in this study, we choose the Mid-Latitude Summer (MLS) as the MODTRAN model atmosphere.

Other data from the study area, including meteorological data (e.g., rainfall, temperature) obtained from the China Meteorological Data Sharing Service System (http://cdc.cma.gov.cn/) of the National Science and Technology Infrastructure, socioeconomic data (e.g., population, cattle stocks) obtained from the national and provincial Statistic Almanac, annual land-use maps (Data Sharing Infrastructure of Earth System Science, http://www.geodata.cn/), and grassland type and soil type data, were collected and collated. To develop a grassland desertification classification system for the study area and interpretation symbols for visual interpretation, a two-week field survey of the study area was performed in August 2013, during which extensive ground data were obtained. These data primarily consisted of spatial-location information, geomorphologic features, sample plot and quadrat information, and grazing and desertification conditions around the sample plot.

### Grading system for Horqin sandy desertification

To monitor the distribution and dynamic changes of grassland desertification and assess policies, a dynamic monitoring system is required with the contributions of advancing technologies and knowledge^6^. However, the formulation of a universal grassland desertification monitoring indicator system is a challenging and ambitious task due to the diverse regional desertification characteristics, technological levels, and cognition levels^43–45^. Dispersed and patchy vegetation cover and sand sheets are the main landscape characteristics of areas undergoing grassland desertification and serve as good visual indicators of environmental changes and the severity of desertification^46^. Based on relevant studies of grassland desertification criteria^47, 48^ and the national standards of Classification Indexes of Degradation, Desertification and Salinization of Natural Grasslands (GB19377-2003), we used the vegetation cover and bare-sand ratio as primary criteria for determining the desertification status. The threshold for the rank of each indicator was set according to the statistical results from the field sample plots and quadrat data, which contain information regarding desertification conditions (classified based on geomorphologic features; the DG and Non-desertified grassland (Non-DG) were distinguished by selecting the apical, stable, and closest regions to the primary grassland community in the Da Qing-Gou Valley Natural Protective Region as the baseline) and the corresponding vegetation coverage data and bare-sand ratios (Table 4). The bare sand ratio was the preferred option to identify desertification because the area with low vegetation coverage caused by non-desertification factors may be overestimated in desertification degree via vegetation coverage^6, 49^. For the sake of contrastive analysis for other studies, the vegetation grading values in Table 4 were retained because they are commonly used in other studies. Furthermore, we identified the following ten land cover classes in the study area (note that shrubland was treated as grassland) combined with land use mapping, grassland type and desertification characters: Farmland, Forestland, Human settlement land, Salinized land, Water, Non-DG land, SlDG, MDG, SeDG and Bare lands^18^.

**Table 4:** Remote sensing grading system of grassland desertification in Horqin.

Grassland desertification intensity classification	Vegetation community characteristics		Bare-sand ratio (%)	Geomorphologic features
	Vegetation composition	Vegetation coverage (%)		
SlDG	Psammophytes become the main accompanying species.	45–60	15–30	Relatively moderate sand, fixed sand dunes.
MDG	Psammophytes become the dominant species.	25–45	30–50	Moderate sand, small blowout pits or semi-fixed sand dunes.
SeDG	Vegetation is very sparse, only a few psammophytes remaining.	<25	>50	Medium and large sand dunes, large blowout pits, semi-mobile or mobile sand dunes.

### Extraction method for indicators

Pixels in remote sensing images of arid and semi-arid environments, particularly in areas undergoing desertification, usually have multiple components^50, 51^. The linear spectral mixture model (LSMM), a SMA that is widely used due to its simplicity, reasonable level of effectiveness and interpretability^52–54^, was selected to calculate the proportions of each target surface feature within a pixel. In the LSMM, the spectral signature of a given pixel is assumed to be a linear combination of each end-member spectrum and its relative abundance, which is represented by Eq. (1):

1 $${\rm{\rho }}({\lambda }_{{i}})=\sum _{j=1}^{{m}}{F}_{j}{\rho }_{j}({\lambda }_{{i}})+ {\mathcal E} ({\lambda }_{{i}})$$

where *j* = 1, 2, … ${m}$ is the pixel component (end-member) and ${i}$ = 1, 2, … ${n}$ is the spectral band. In addition, the number of end-members was constrained by the dimensionality of the satellite images (${m}$ ≤ ${n}$ + 1)^55, 56^; ${\rm{\rho }}({\lambda }_{{i}})\,$ is the composite reflectance of the mixed pixels in band ${i}$; ${\rho }_{j}({\lambda }_{{i}})$ is the reflectance of the end-member *j* in band ${i}$; ${F}_{j}$ refers to the abundance of the end-member $j$ in the pixel (a parameter to be estimated); and ${\mathcal E} ({\lambda }_{{i}})$ is the difference between the actual and modelled reflectance. In addition, ${F}_{j}$ represents the best-fit coefficient that minimizes the RMS error, which is obtained using Eq. (2):

2 $$RMS=\sqrt{\frac{({\sum }_{{i}=1}^{{n}}{\varepsilon }^{2}({\lambda }_{{i}}))}{{n}}}$$

where ${n}$ is the number of bands and ${\mathcal E} ({\lambda }_{{i}})$ is the residual term in band ${i}$.

**Figure 5:**
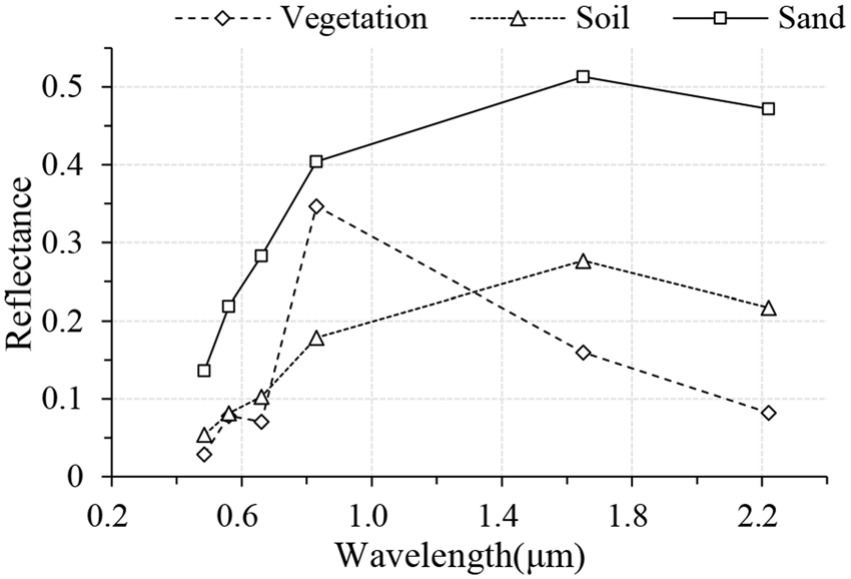
Averaged reflectances of endmembers.

Appropriate selection of the end-members representing the surface components is crucial for the success of LSMM^57^. LSMM with inadequate end-members easily results in the misdivision of the un-modelled land cover components into modelled components (end-members), while many end-members become sensitive to noise^58^. The endmember should be representative and an effective component of most of the images within the pixel to produce physically realistic proportions, minimize the RMS error and force the derived fractions of the end-members constrained to non-negative values with a sum of 1^55, 59^. In this study, we conducted a comprehensive field investigation to identify the optimal or most representative surface types and identify preferable locations for the development of a spectral library from the Landsat images^54, 60^. Based on the field investigations, “pure” spectral end-members of vegetation, bare sand and bare soil were extracted using the minimum noise fraction (MNF) and the Pixel Purity Index algorithm (PPI) by iteratively testing different endmember combinations (Fig. 5), which is a common approach for selecting end-members and is available through ENVI^61–64^.

To focus on desertification information and simplify the process of endmember selection, the normalized difference infrared index $({\rm{NDII}}=({\rho }_{819}-{\rho }_{1649})/({\rho }_{819}+{\rho }_{1649})$, where *ρ*_819_ and *ρ*_1649_ are the reflectances in the electromagnetic spectrum at 819 nm and 1649 nm, respectively)^65^, was used to mask farmland; the Tasseled Cap (TC), band5 and NDVI were used to mask forestland; the modified normalized differential water index (MNDWI) was used to mask water areas; and the TC_1_ (the first band of the TC) and MNDWI were used to mask salinized land. Next, the LSMM algorithm was applied to the masked image, and a grading desertification map was produced from the bare sand fraction map using the decision-tree method based on the grading system (see Supplementary Figure S2 for details).

### Spatio-temporal change detection

To reveal the mechanisms responsible for grassland desertification spatio-temporal dynamic changes, raster calculation available in the ArcGIS and change detection and decision tree available in ENVI were performed. Before this perform, the farmland, forestland, human settlement land, water, salinized land, and bare land classes were merged as non-grassland. Then, the following criteria were used: an area with an increase in the degree of desertification is designated as ‘Deteriorated’ (e.g., a change from SlDG to MDG); an area with a cross-level increase in the degree of desertification is designated as ‘Seriously Deteriorated’ (e.g., a change from SlDG to SeDG). Similarly, the ‘Reversed’ and ‘Significantly Reversed’ classifications were defined in the same way. An area without any changes in the degree of desertification is referred to as ‘stable’, except where a change from grassland to non-grassland or vice versa occurs.

### Field survey

**Figure 6:**
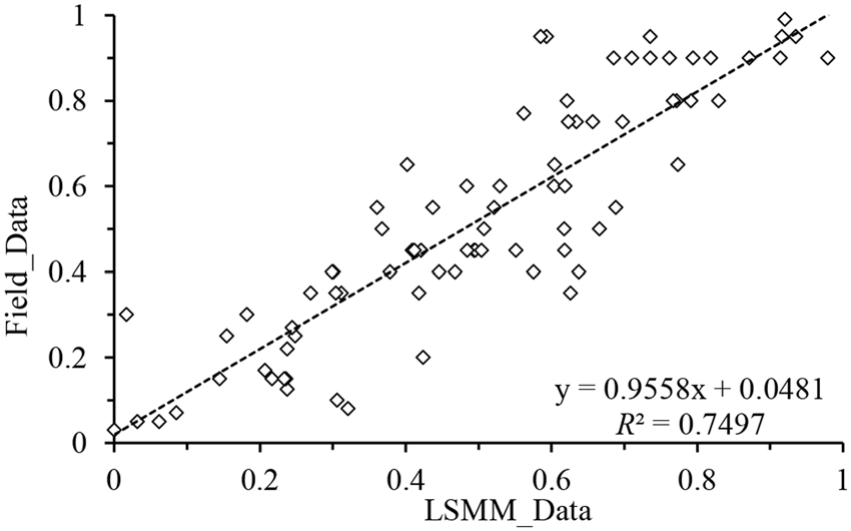
Scatter plot correction between the field-measured and LSMM-estimated vegetation fractions in 2013.

To develop interpretation symbols, a grading system consistent with conditions in the HSL, and field truth data as references for validation, a field survey was conducted on 5–20 August, 2013. The dates of the field survey nearly coincided with of the dates of the Landsat images, which ensured the reliability of the interpretation symbols, grading system and result validation. In this article, 125 plots were selected that contained herbs and low shrubs (measuring 1 × 1 m) or shrubs (measuring 10 × 10 m). Plots were georeferenced using GPS, and the vegetation canopy was orthogonally projected to the ground to estimate the vegetation coverage. After excluding the plots used for end-member selection and for interpretation symbols development, 81 plots remained for validation. The accuracy was estimated from a scatter plot correction to compare the field vegetation cover in each plot with the pixel values extracted from the vegetation fraction image (derived from LSMM) by using the extract-value-to-points function via the ArcGIS software (Fig. 6).

## Electronic supplementary material

Supplementary info
