# Supplementary material for: Historical grassland desertification changes in the Horqin Sandy Land, Northern China (1985–2013)
Source: Sci Rep. 2017 Jun 7;7:3009. doi: 10.1038/s41598-017-03267-x (PMC5462796; doi:10.1038/s41598-017-03267-x)

Historical grassland desertification changes in the Horqin Sandy Land, Northern China (1985-2013)

Jinya Li^a,b^, Bin Xu^b^, *, Xiuchun Yang^b^, Zhihao Qin^b^, Lina Zhao^c^, Yunxiang Jin^b^, Fen Zhao^d^, Jian Guo^b^

**Supplementary Table S1. Basic information of used Landsat TM/ETM+/OLI.**

| **Path** | **Row** | **1985s** | **1992** | **2001** | **2013** |
| --- | --- | --- | --- | --- | --- |
| 119 | 29 | 1985.08.07 | 1992.08.26 | 2001.08.11 | 2013.08.20 |
| 119 | 30 | 1985.08.07 | 1992.08.26 | 2001.08.11 | 2013.07.3 |
| 120 | 29 | 1985.08.30 | 1992.09.02 | 2001.08.10 | 2013.09.12 |
| 120 | 30 | 1986.09.18 | 1992.09.02 | 2001.08.10 | 2013.09.12 |
| 120 | 31 | 1985.07.31 | 1992.09.02 | 2001.08.10 | 2013.09.12 |
| 121 | 28 | 1985.08.21 | 1992.07.23 | 2001.09.10 | 2013.09.03 |
| 121 | 29 | 1985.08.21 | 1992.07.23 | 2001.09.10 | 2013.09.03 |
| 121 | 30 | 1985.08.21 | 1992.07.23 | 2001.09.10 | 2013.09.03 |
| 121 | 31 | 1985.09.06 | 1992.08.24 | 2001.09.10 | 2013.09.03 |
| 122 | 28 | 1985.08.12 | 1992.09.16 | 2001.09.09 | 2013.09.10 |
| 122 | 29 | 1985.09.13 | 1992.09.16 | 2001.09.09 | 2013.09.10 |
| 122 | 30 | 1985.08.28 | 1992.09.16 | 2001.09.09 | 2013.08.09 |
| 123 | 29 | 1985.09.04 | 1993.09.10 | 2001.06.28 | 2013.09.12 |

**Supplementary Figure S2. Flow chart.** The map was generated by Edraw Max software (SHENZHEN EDRAW SOFTWARE CO.,LTD., Shenzhen, China, http://www.edrawmax.com).


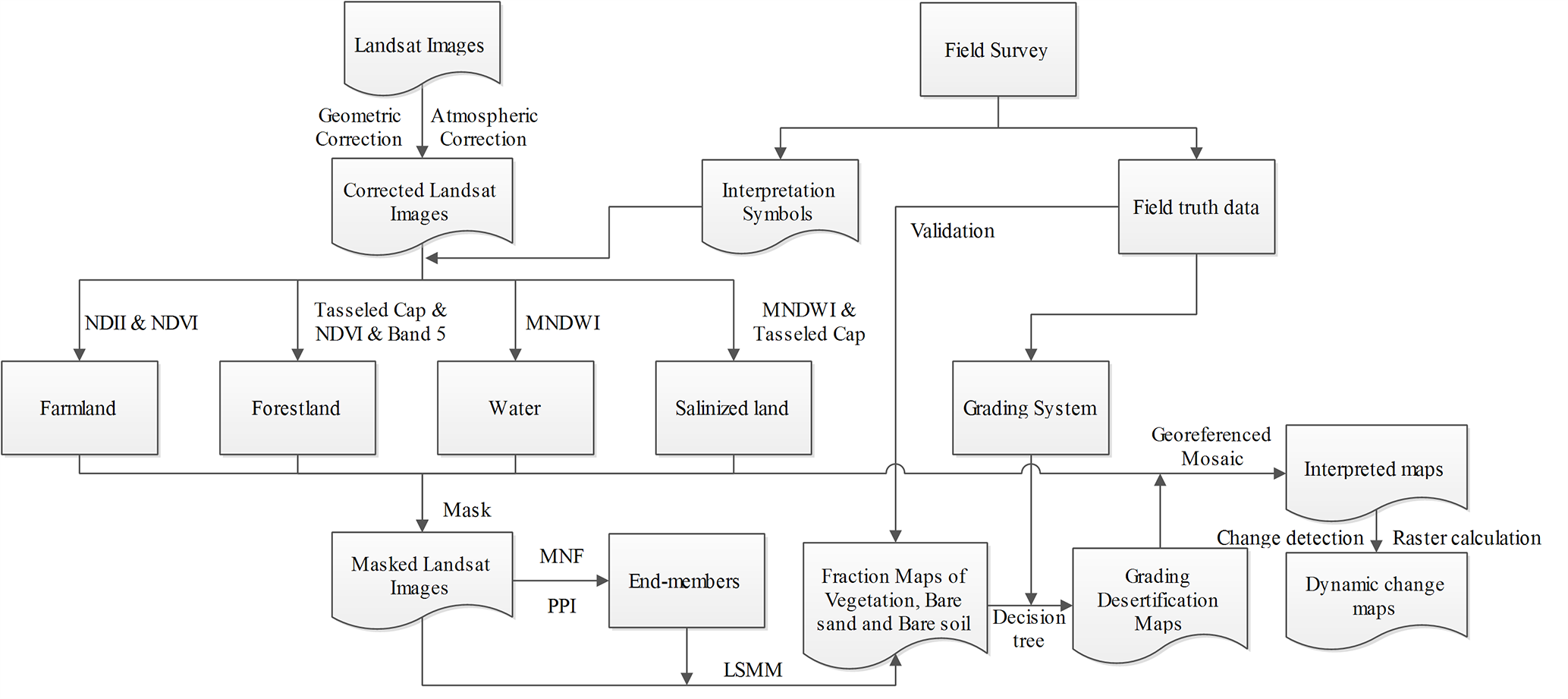

Supplement: Supplementary file 1 — Supplementary info [file 41598_2017_3267_MOESM1_ESM.doc]
